# The “5E” hierarchical nursing strategy: Improving catheter outcomes, patient satisfaction, and self-management in oncology day-care settings

**DOI:** 10.1097/MD.0000000000046354

**Published:** 2025-12-26

**Authors:** Yanyan Chen, Lunlan Li

**Affiliations:** a School of Nursing, Anhui Medical University, Hefei, Anhui, China; b Day-Care Ward, The First Affiliated Hospital of Anhui Medical University, Hefei, Anhui, China; c Human Resource Department, The First Affiliated Hospital of Anhui Medical University, Hefei, Anhui, China.

**Keywords:** 5E nursing strategy, day-care model, hierarchical management, oncology nursing, peripheral venous catheter

## Abstract

Peripheral venous catheters (PVCs) are commonly used in day-care oncology settings, but catheter occlusion and related complications can negatively affect both patient experience and nursing quality. Therefore, exploring effective nursing strategies to minimize complications, enhance satisfaction, and strengthen patients’ self-management skills is of significant clinical value. This retrospective controlled study involved 286 oncology patients receiving PVC infusions in the day ward between March and August 2021. Based on admission period, patients were assigned to 2 groups: 142 patients admitted from March to May received routine nursing care (control group), while 144 patients admitted from June to August were managed under a hierarchical “5E” nursing strategy (observation group). The 5E strategy comprised Evaluation, Education, Environment optimization, Engineering control, and Execution enhancement. Outcomes compared between groups included baseline characteristics, catheter occlusion rate, mean indwelling time, nursing satisfaction, self-management ability, and incidence of adverse events. Baseline characteristics were comparable (*P* > .05). The observation group demonstrated a lower occlusion rate (4.9% vs 13.4%, *P* = .014), longer average indwelling duration (4.85 ± 1.13 vs 4.32 ± 1.05 days, *P* < .001), and higher nursing satisfaction (95.1% vs 85.2%, *P* = .008). Self-management scores were also significantly higher (87.6 ± 6.8 vs 80.9 ± 7.5, *P* < .001), particularly in execution ability and symptom recognition. Additionally, adverse event incidence was lower in the observation group (6.3% vs 14.1%, *P* = .029). The hierarchical management–based “5E” nursing strategy effectively reduces PVC occlusion and adverse events, extends indwelling time, and improves both satisfaction and self-management among oncology day-care patients. This approach shows strong potential for wider clinical adoption.

## 1. Introduction

In recent years, with the continuous advancement of cancer treatment concepts and the optimized allocation of medical resources, the day-care treatment model has rapidly developed in oncology-specialized and general hospitals across China.^[1–3]^ This model not only shortens hospital stays and reduces healthcare costs but also improves bed turnover rates and enhances patient experience, making it particularly suitable for cancer patients requiring frequent infusions or cyclical therapies. Under this paradigm, peripheral venous catheters (PVCs) have been widely used in chemotherapy, targeted therapy, nutritional support, and traditional Chinese medicine infusions, due to their advantages of reduced puncture frequency, ease of infusion, and minimal trauma to patients.^[4,5]^ However, factors such as high patient turnover, fast-paced workflows, and limited patient self-care knowledge in day wards may increase the risk of catheter occlusion and related complications, thereby affecting treatment efficacy and adherence.^[6–9]^ Thus, developing a safe, systematic, and feasible nursing management strategy tailored for the day-care environment has become an urgent issue in oncology nursing.

Existing studies have shown that structured and process-oriented interventions can reduce catheter-related complications and improve patient experience.^[10–13]^ For example, “closed-loop management” strategies emphasize standardized flushing and monitoring procedures, while health education frameworks such as the Knowledge-attitude-practice model applied in oncology settings have improved complication control and patient satisfaction.^[14,15]^ More recent international studies on PVC management also highlight challenges in device securement, complication prevention, and patient involvement in self-care.^[16–18]^ However, most available approaches focus on isolated interventions or single procedures and lack comprehensive integration of multidimensional risk management elements suitable for the fast-paced, high-turnover day-care context.

Compared with the knowledge-attitude-practice model, which mainly emphasizes patient education, and closed-loop management, which focuses on procedural standardization, the “5E” nursing strategy provides a more integrated framework. By combining hierarchical management with elements such as environmental optimization and execution reinforcement, the 5E model not only ensures technical standardization but also enhances patient engagement and self-management. This holistic and layered approach is more suitable for the complex characteristics of oncology day-care nursing. The 5E strategy – evaluation, education, environment, engineering, and execution – originates from clinical pathway design and quality improvement concepts. It advocates a full-cycle nursing model that integrates assessment, education, environmental optimization, technical standardization, and quality enforcement. While the framework has been preliminarily applied in intensive care units, perioperative care, and chronic disease management, studies on its use in oncology day-care PVC management remain scarce.

This study is the first to implement the 5E strategy in PVC management among oncology day-care patients, combined with hierarchical nursing principles to establish personalized care plans. By retrospectively analyzing nursing records of 286 patients, we compared the outcomes of 5E nursing versus routine care in terms of catheter occlusion, indwelling time, nursing satisfaction, self-management, and adverse events. Unlike previous studies that emphasized procedural refinement alone, our work highlights the integration of patient engagement and behavioral intervention, aiming to construct a systematic, risk-based, and promotable nursing model for day-care oncology settings.

## 2. Method

### 2.1. Study subjects and grouping

This research was approved by the Ethics Committee of The First Affiliated Hospital of Anhui Medical University. This retrospective controlled study included a total of 286 oncology patients who received PVC infusion therapy in the day ward of our hospital from March 1 to August 31, 2021. Patients were grouped based on their admission dates: 142 patients admitted between March 1 and May 31 were assigned to the control group, and 144 patients admitted between June 1 and August 31 were assigned to the observation group. Inclusion criteria were: age ≥ 18 years, complete clinical data, agreement to receive PVC infusion therapy, and willingness to comply with relevant nursing interventions. Exclusion criteria included severe organic disease, cognitive impairment, or incomplete infusion cycle.

### 2.2. Nursing interventions

The control group received standard PVC care, including routine assessment of the puncture site, flushing and sealing techniques, and complication management. The observation group received the “5E” nursing strategy in addition to routine care. This strategy consists of 5 components:

Evaluation: systematic assessment of baseline status, vascular condition, and self-care ability prior to intervention.

Education: delivery of health education via printed manuals and individualized guidance to enhance patient awareness and initiative.

Environment: optimization of treatment space in terms of cleanliness, quietness, and comfort.

Engineering: standardization of catheter selection, fixation, flushing, and sealing procedures.

Execution: establishment of a process-oriented quality control system to enhance consistency and adherence in nursing execution.

### 2.3. Outcome measures

The following clinical indicators were compared between the 2 groups:

1. General information: age, sex, height, weight, blood pressure, heart rate, blood glucose, smoking history, primary cancer type, and type of infused drugs.
2. Incidence of catheter occlusion.
3. Average catheter indwelling time.
4. Patient satisfaction with nursing care, assessed using a self-designed questionnaire (total score: 100, satisfaction defined as ≥ 80).
5. Self-management ability, including 4 dimensions: information acquisition, symptom recognition, problem-solving ability, and execution ability (total score: 100).
6. Incidence of adverse events, including infusion site leakage, redness, pain, and local induration.

### 2.4. Scoring and data collection

Nursing satisfaction and self-management ability were evaluated by trained nurses via face-to-face interviews or telephone follow-ups on the day of discharge. Catheter occlusion and adverse events were recorded by the responsible nurse based on patient records and ward observations. All raw data were independently entered and cross-verified by 2 researchers to ensure accuracy and consistency.

### 2.5. Statistical analysis

Statistical analyses were performed using SPSS version 26.0 (IBM Corporation, Armonk). Continuous variables were expressed as mean ± standard deviation ($\bar{x}\pm s$) and compared using independent samples *t* tests. Categorical variables were expressed as frequencies and percentages, and comparisons were made using Chi-square (χ^2^) tests. A *P*-value < .05 was considered statistically significant.

## 3. Result

### 3.1. Comparison of baseline characteristics

A total of 286 oncology patients who received PVC infusion in the day ward of our hospital between March 1 and August 31, 2021, were included in this study. Based on the order of admission, 142 patients admitted from March 1 to May 31 were assigned to the control group, and 144 patients admitted from June 1 to August 31 were assigned to the observation group. Baseline characteristics compared between the 2 groups included cancer type, type of infused drugs, height, weight, heart rate, systolic blood pressure, diastolic blood pressure, smoking history, and fasting blood glucose. There were no statistically significant differences in any of these variables between the 2 groups (*P* > .05), indicating good comparability (see Table 1).

**Table 1:** Comparison of general characteristics between the 2 groups (n, %/$\bar{x}\pm s$).

Variable	Control group (n = 142)	Observation group (n = 144)	χ^2^/*t* value	*P*-value
Type of cancer				
Lung cancer	42 (29.6%)	37 (25.7%)	χ^2^=1.064	.786
Cervical cancer	39 (27.5%)	47 (32.6%)		
Gastric cancer	31 (21.8%)	31 (21.5%)		
Colorectal cancer	30 (21.1%)	29 (20.1%)		
Type of infused drug				
Antibiotics	22 (15.5%)	23 (16.0%)	χ^2^=0.059	1
Nutritional solution	23 (16.2%)	24 (16.7%)		
Traditional Chinese medicine	36 (25.4%)	35 (24.3%)		
Targeted therapy	30 (21.1%)	30 (20.8%)		
Chemotherapy	31 (21.8%)	32 (22.2%)		
Height (cm)	163.7 ± 6.8	164.1 ± 6.5	*t* = 0.489	.625
Weight (kg)	60.4 ± 9.5	61.1 ± 9.2	*t* = 0.621	.535
Heart rate (bpm)	75.6 ± 8.1	76.2 ± 8.4	*t* = 0.588	.557
Systolic BP (mm Hg)	126.4 ± 11.5	125.9 ± 10.9	*t* = 0.404	.686
Diastolic BP (mm Hg)	77.2 ± 8.3	76.8 ± 8.0	*t* = 0.407	.684
Smoking history (yes/no)	41 (28.9%)/101 (71.1%)	39 (27.1%)/105 (72.9%)	χ^2^=0.126	.723
Fasting blood glucose (mmol/L)	5.67 ± 0.98	5.63 ± 1.01	*t* = 0.339	.735

### 3.2. Comparison of catheter occlusion rates

**Figure 1.**
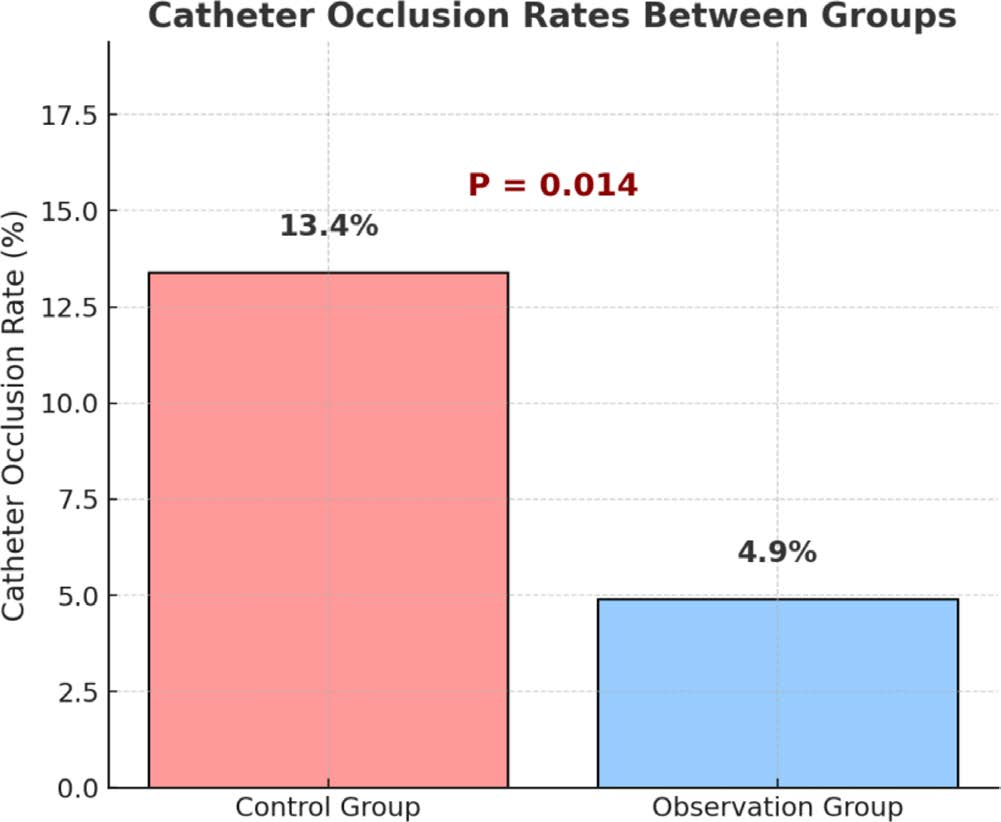
Catheter occlusion rates between groups.

During the nursing intervention period, the incidence of PVC occlusion was monitored and recorded in both groups. The results showed that the occlusion rate in the observation group was 4.9% (7/144), significantly lower than 13.4% (19/142) in the control group. The difference was statistically significant (χ^2^ = 6.008, *P* = .014), as shown in Figure 1. These findings suggest that the implementation of the “5E” nursing strategy – comprising evaluation, education, environment optimization, engineering (including both control of standardized procedures and optimization of techniques), and execution enhancement – under hierarchical management significantly reduces the risk of catheter occlusion and contributes to the safety and stability of infusion pathways.

### 3.3. Comparison of average catheter indwelling time

**Figure 2.**
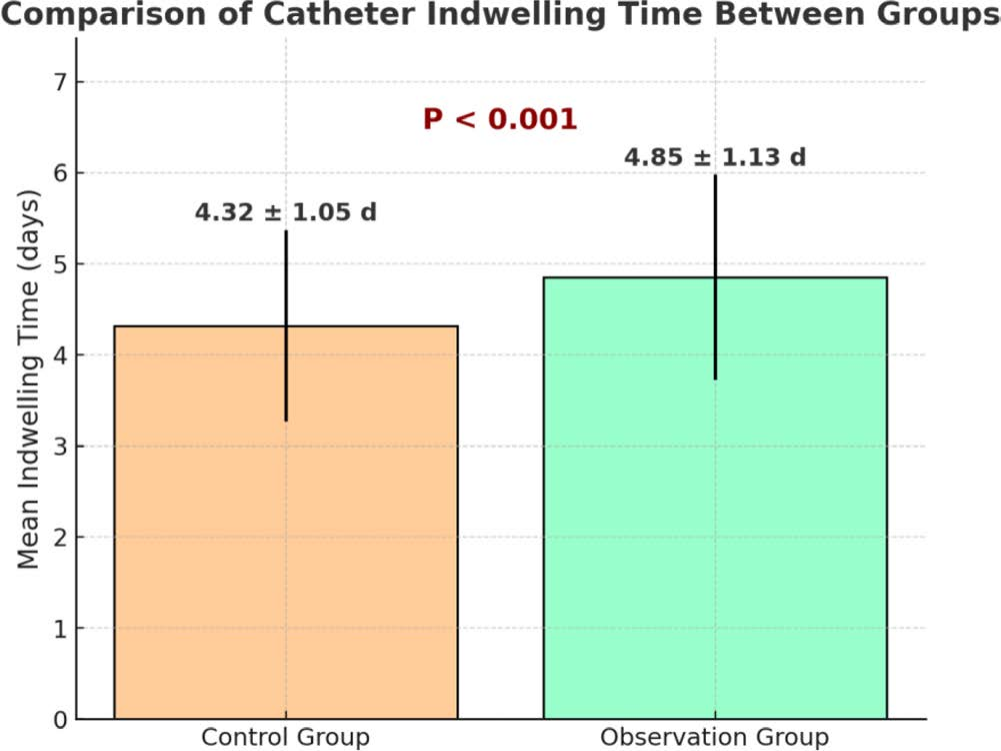
Comparison of catheter indwelling time between groups.

To further evaluate the effectiveness of the “5E” nursing strategy in catheter maintenance, the average indwelling time of PVCs was compared between the 2 groups. As shown in Figure 2, the observation group had a significantly longer average indwelling time (4.85 ± 1.13 days) compared to the control group (4.32 ± 1.05 days). The difference was statistically significant (*t* = 3.996, *P* < .001), indicating that the hierarchical “5E” nursing model is effective in prolonging catheter indwelling time and optimizing infusion pathway management.

### 3.4. Comparison of patient satisfaction with nursing care

**Figure 3.**
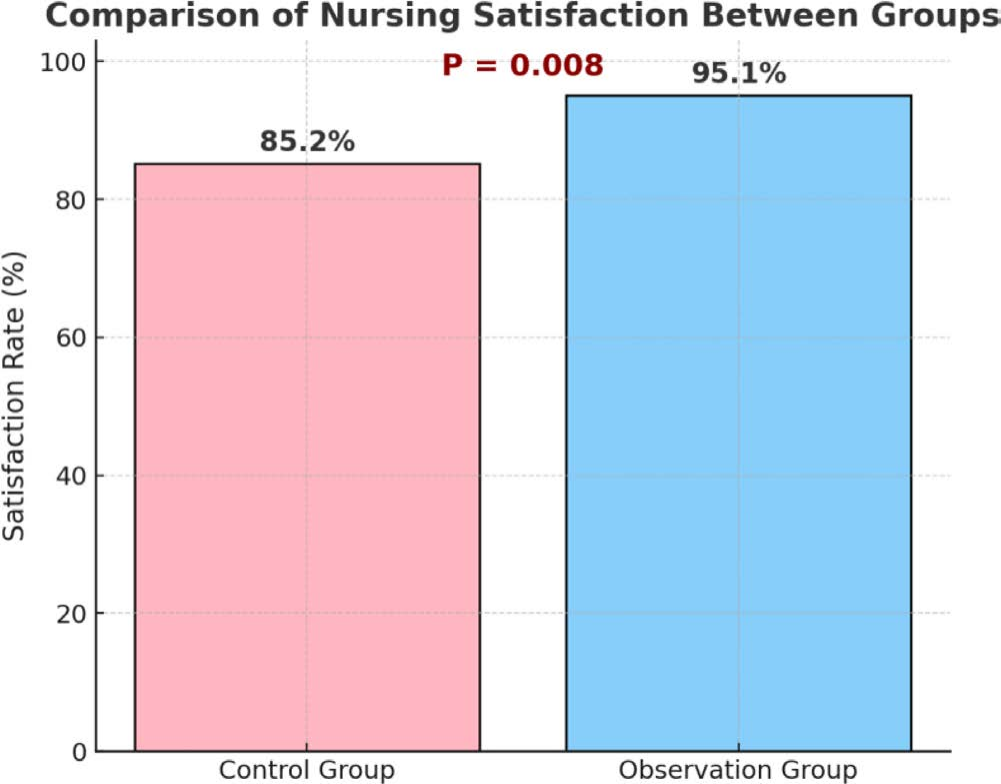
Comparison of nursing satisfaction between groups.

To assess the impact of nursing strategies on patient-perceived experience, the research team administered a self-designed nursing satisfaction questionnaire to patients upon discharge. Satisfaction was evaluated across multiple dimensions, including nursing attitude, technical standardization, communication initiative, comfort, and operational accuracy. A standardized scoring system was used, with a total score ≥ 80 points defined as “satisfied.” As shown in Figure 3, the satisfaction rate in the observation group was 95.1% (137/144), significantly higher than 85.2% (121/142) in the control group. The difference was statistically significant (χ^2^ = 7.032, *P* = .008), indicating that implementation of the “5E” nursing model under hierarchical management effectively enhances overall patient satisfaction and promotes positive nurse–patient interaction and trust.

### 3.5. Comparison of self-management ability scores

**Figure 4.**
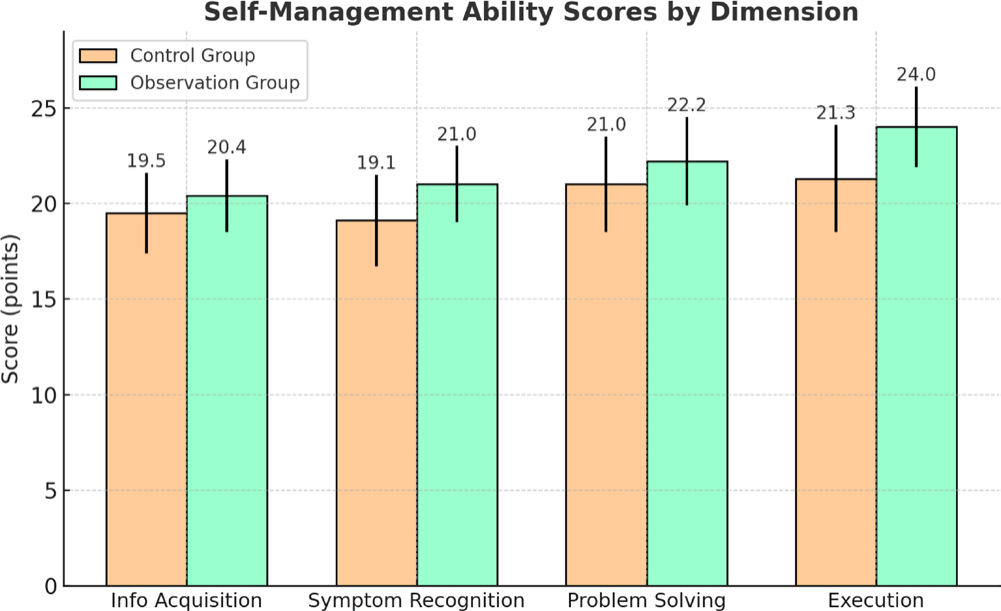
Self-management ability scores by dimension.

To evaluate the impact of the “5E” nursing intervention on patients’ self-care ability after discharge, a self-management assessment was conducted for both groups before discharge. The assessment covered 4 dimensions: information acquisition, symptom recognition, problem-solving ability, and execution ability, with each dimension scored independently and the total score set at 100 points. As shown in Table 2 and Figure 4, the observation group achieved a significantly higher total score (87.6 ± 6.8) compared to the control group (80.9 ± 7.5), with the difference being statistically significant (*t* = 7.315, *P* < .001). Notably, the most significant improvements were observed in execution ability and symptom recognition (*P* < .01). These results suggest that the “5E” nursing model, through active education, behavioral guidance, and execution reinforcement, can substantially enhance patients’ disease management capabilities, thus improving self-care efficiency and safety in the context of day-care treatment.

**Table 2:** Comparison of self-management ability scores between groups ($\bar{x}\pm s$).

Dimension	Control group (n = 142)	Observation group (n = 144)	*t* value	*P*-value
Information acquisition	19.5 ± 2.1	20.4 ± 1.9	3.767	<.001*
Symptom recognition	19.1 ± 2.4	21.0 ± 2.0	6.713	<.001*
Problem-solving ability	21.0 ± 2.5	22.2 ± 2.3	4.041	<.001*
Execution ability	21.3 ± 2.8	24.0 ± 2.1	8.323	<.001*
Total score	80.9 ± 7.5	87.6 ± 6.8	7.315	<.001*

* *P* < .05.

### 3.6. Comparison of adverse event incidence

**Figure 5.**
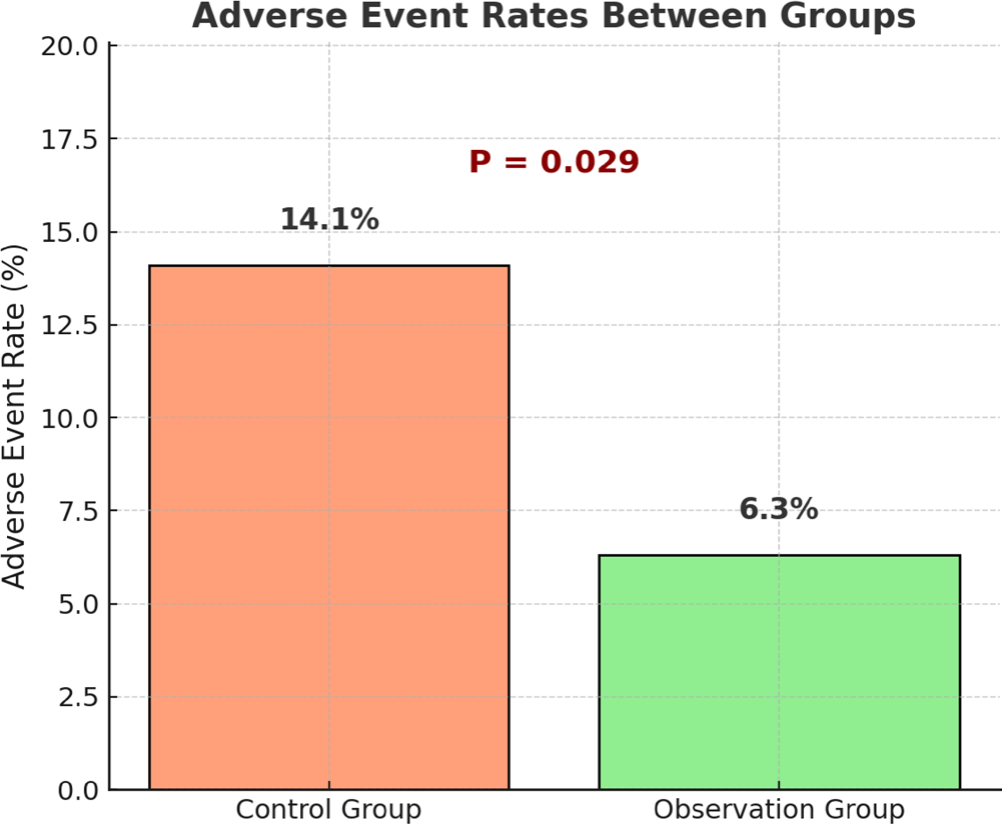
Adverse event rates between groups.

This study further evaluated the effect of the “5E” nursing strategy on the prevention of infusion-related complications. Common adverse events, excluding catheter occlusion, were recorded, including infusion site leakage, skin redness, pain, and local induration. As shown in Figure 5, the incidence of adverse events was 14.1% (20/142) in the control group and 6.3% (9/144) in the observation group, with a statistically significant difference between the groups (χ^2^ = 4.753, *P* = .029). These findings indicate that the “5E” strategy – through engineering optimization, standardized procedure reinforcement, environmental improvement, and risk-focused education – effectively reduces the occurrence of infusion-related complications and enhances the safety of intravenous therapy.

## 4. Discussion

With the widespread adoption of day-care treatment models in oncology nursing, the safety and efficiency of PVCs have received increasing attention in clinical practice.^[19]^ Given the fast-paced workflow and frequent patient turnover in day-care settings, catheter occlusion and related complications not only reduce infusion efficiency but may also lead to treatment delays, increased patient anxiety, and even medical disputes.^[20]^ Therefore, there is a pressing need to explore a structured, operable, and scenario-adapted nursing strategy to improve patient safety, satisfaction, and self-management during intravenous therapy. Based on the concept of graded nursing management, this study implemented the “5E” nursing strategy – comprising evaluation, education, environment, engineering, and execution – targeting multiple levels and processes to enhance infusion safety, comfort, and compliance in patients with cancer undergoing day-care treatment.

A total of 286 patients were enrolled and grouped by admission period to compare the clinical outcomes between those receiving routine care and those receiving the 5E-based intervention. No significant differences were found in baseline characteristics between groups, ensuring comparability and the reliability of outcome measures.

Regarding catheter occlusion, the incidence in the intervention group was significantly lower than in the control group (4.9% vs 13.4%, *P* = .014). This is consistent with previous studies that showed structured nursing pathways could effectively reduce occlusion rates.^[21]^ The innovation of the 5E strategy lies in its integration of “engineering” and “execution” dimensions, including standardized flushing protocols and real-time tracking of infusion paths, leading to a more systematic and measurable nursing process. These enhancements contributed to a meaningful reduction in occlusion events, underscoring the innovation of this study in preventive nursing mechanisms.

The intervention group also had significantly longer catheter indwelling time (4.85 ± 1.13 vs 4.32 ± 1.05 days, *P* < .001), suggesting improved catheter maintenance and durability. Similar findings have been reported in studies applying full-cycle infusion management models, which moderately extended catheter lifespan.^[22]^ However, those interventions focused primarily on operational protocols, whereas the current study incorporated environmental optimization and patient education modules, thereby enhancing patient cooperation and maximizing the nursing effect. Although the increase was only about 0.5 days, this modest extension has practical significance, as it reduces repeated venipuncture, alleviates patient discomfort, decreases nursing workload and material use, and supports continuity of therapy, ultimately benefiting both patients and healthcare systems.

In terms of nursing satisfaction, the intervention group reached 95.1%, significantly higher than the control group’s 85.2% (*P* = .008). Patient satisfaction is a critical subjective indicator of nursing quality, especially in high-turnover settings like day-care wards. High satisfaction not only reflects care quality but also promotes adherence to ongoing treatment. The improvements observed in this study may be attributed to personalized education and environmental enhancements – such as comfort optimization of infusion areas, improved privacy, and streamlined service processes – which alleviated patient discomfort and anxiety, thereby contributing to higher satisfaction scores.

Self-management ability is a key indicator of health literacy and post-discharge quality of life, particularly important in cancer patients requiring follow-up care at home. The intervention group achieved significantly higher total and subdomain scores (87.6 ± 6.8 vs 80.9 ± 7.5, *P* < .001), with the most notable improvements in execution and symptom recognition abilities. These results align with existing research in chronic disease management that emphasizes structured patient education. However, our study further adopted an “engineering-behavior synergy” model, embedding self-management training within process control and execution reinforcement. This approach effectively facilitated a transition from passive compliance to active self-care, highlighting a systems-level innovation in nursing methodology.^[23]^

Furthermore, the incidence of other adverse events (e.g., infiltration, redness, pain) was also significantly lower in the intervention group (6.3% vs 14.1%, *P* = .029). This suggests that the 5E strategy offers a broad-spectrum risk control benefit – not limited to occlusion – but covering multiple dimensions across the infusion care continuum. The approach reflects a holistic nursing model characterized by full-process risk management and multidisciplinary integration. Moreover, the feasibility of implementing the 5E nursing strategy in different countries and healthcare systems warrants further consideration. Although the core components of the model – comprehensive evaluation, patient education, environmental optimization, engineering standardization, and execution reinforcement – are conceptually universal, their practical application may vary according to local healthcare infrastructure, nurse-to-patient ratios, cultural norms, and patient health literacy. For instance, in high-resource healthcare systems, the model could be seamlessly integrated into existing quality improvement frameworks and supported by advanced training programs. In contrast, in resource-limited settings, simplified adaptations focusing on education and execution reinforcement might be more realistic and cost-effective. Therefore, while our findings provide a promising framework for improving PVC outcomes in oncology day-care settings, further multicenter and cross-national studies are needed to validate its applicability across diverse healthcare contexts.

In contrast to most existing studies that target single outcomes such as occlusion or satisfaction, our study is the first to simultaneously incorporate 5 key nursing indicators – catheter patency, usage duration, self-management, satisfaction, and complication rates – into a unified intervention system under the day-care oncology context. The integration of these metrics through the 5E framework demonstrated strong systemic, structured, and scalable advantages, adding both clinical relevance and methodological innovation to the literature.

Nonetheless, several limitations should be acknowledged. First, the single-center retrospective design may limit generalizability. Second, both nursing satisfaction and self-management scores were based on patient self-report, which may be subject to subjective bias despite standardized questionnaires. Third, the short follow-up period precludes evaluation of long-term adherence and complication control. Fourth, patient grouping was based on admission period (March–May vs June–August), which may introduce potential time bias due to seasonal variation or changes in nursing personnel. To minimize this influence, the hospital maintained consistent staffing, standardized nursing protocols, and unified quality control procedures throughout the study period; however, the possibility of residual temporal effects cannot be completely excluded. Future studies with prospective, multicenter designs and extended follow-up are warranted to validate and expand upon our findings.

In conclusion, the 5E nursing strategy based on graded management significantly reduced catheter occlusion and adverse event rates while improving catheter utilization time, patient satisfaction, and self-management capability. This model is particularly suitable for the fast-paced requirements of day-care oncology settings and has promising potential for broader clinical implementation. Future work should focus on multicenter validation and further optimization of the 5E intervention pathway.

## 5. Conclusion

This study, grounded in the concept of graded management, developed and implemented the “5E” nursing strategy and systematically evaluated its effectiveness in managing PVCs among oncology patients in a day-care setting. The results demonstrated that the 5E intervention significantly reduced catheter occlusion rates, prolonged catheter indwelling time, improved patient satisfaction and self-management capabilities, and decreased the incidence of adverse events. Compared with traditional nursing models, this strategy integrates assessment, education, environmental optimization, technical standardization, and execution reinforcement to form a structured, process-oriented, and individualized closed-loop nursing system. It is particularly suitable for high-paced clinical scenarios characterized by frequent care transitions and multiple risk points. As an innovative and potentially generalizable nursing model, further validation through multicenter studies is recommended to confirm its replicability and long-term benefits. However, as this was a single-center, retrospective study, the findings should be interpreted with caution. Further prospective, multicenter investigations are warranted to validate the replicability and long-term benefits of the 5E nursing model across diverse clinical settings.

## Author contributions

**Conceptualization:** Yanyan Chen, Lunlan Li.

**Data curation:** Yanyan Chen, Lunlan Li.

**Funding acquisition:** Yanyan Chen, Lunlan Li.

**Investigation:** Yanyan Chen, Lunlan Li.

**Methodology:** Yanyan Chen.

**Supervision:** Lunlan Li.

**Validation:** Yanyan Chen, Lunlan Li.

**Visualization:** Yanyan Chen, Lunlan Li.

**Writing – original draft:** Yanyan Chen, Lunlan Li.

**Writing – review & editing:** Yanyan Chen, Lunlan Li.

## References

[1] Mo H Zhong R Ma F. Chinese expert consensus on an innovative patient-centered approach to diagnosis and treatment of cancer. Cancer Innov. 2024;3:e137. 39081932 10.1002/cai2.137 PMC11287348

[2] Che G. Current situation and strategy of day surgery in patients with lung cancer by enhanced recovery after surgery (In Chinese). Zhongguo Fei Ai Za Zhi. 2020;23:1–4. 31948531 10.3779/j.issn.1009-3419.2020.01.01 PMC7007390

[3] China Tumor Day Diagnosis and Treatment Cooperative Group. [Expert consensus on day care cancer diagnosis and treatment in China (2022 Edition)] (In Chinese). Zhonghua Zhong Liu Za Zhi. 2022;44:307–20. 35448918 10.3760/cma.j.cn112152-20220223-00123

[4] Ray-Barruel G Xu H Marsh N Cooke M Rickard CM. A survey of infection prevention and vascular access device management: a global perspective of 69 countries. J Hosp Infect. 2019;103:223–9.31054935

[5] Alexandrou E. International prevalence of the use of peripheral intravenous catheters. J Vasc Access. 2020;21:456–61.31680607

[6] Wesselius HM van den Ende ES Alsma J; “Onderzoeks Consortium Acute Geneeskunde” Acute Medicine Research Consortium. Quality and quantity of sleep and factors associated with sleep disturbance in hospitalized patients. JAMA Intern Med. 2018;178:1201–8. 30014139 10.1001/jamainternmed.2018.2669 PMC6142965

[7] Kudchadkar SR Berger J Patel R. Non-pharmacological interventions for sleep promotion in hospitalized children. Cochrane Database Syst Rev. 2022;6:CD012908. 35703367 10.1002/14651858.CD012908.pub2 PMC9199068

[8] Vincent JL. The continuum of critical care. Crit Care. 2019;23(Suppl 1):122. 31200740 10.1186/s13054-019-2393-x PMC6570628

[9] Griffiths P Maruotti A Recio Saucedo A; Missed Care Study Group. Nurse staffing, nursing assistants and hospital mortality: retrospective longitudinal cohort study. BMJ Qual Saf. 2019;28:609–17. 10.1136/bmjqs-2018-008043 PMC6716358 30514780

[10] Millington SJ Hendin A Shiloh AL Koenig S. Better with ultrasound: peripheral intravenous catheter insertion. Chest. 2020;157:369–75. 31654617 10.1016/j.chest.2019.04.139

[11] Bell T O’Grady NP. Prevention of central line-associated bloodstream infections. Infect Dis Clin North Am. 2017;31:551–9. 28687213 10.1016/j.idc.2017.05.007 PMC5666696

[12] Moureau N. Hydrophilic biomaterial intravenous hydrogel catheter for complication reduction in PICC and midline catheters. Expert Rev Med Devices. 2024;21:207–16. 38445649 10.1080/17434440.2024.2324885

[13] Golling E van de Mortel T Barr N Zimmerman PA. Pre-hospital peripheral intravenous catheter insertion practice: an integrative review. Australas Emerg Care. 2023;26:105–12. 36117094 10.1016/j.auec.2022.08.006

[14] Paterson RS Schults JA Slaughter E. Review article: peripheral intravenous catheter insertion in adult patients with difficult intravenous access: a systematic review of assessment instruments, clinical practice guidelines and escalation pathways. Emerg Med Australas. 2022;34:862–70. 36038953 10.1111/1742-6723.14069 PMC9804581

[15] Xu HG Keogh S Ullman AJ. Implementation frameworks, strategies and outcomes used in peripheral intravenous catheter studies: a systematic review. J Clin Nurs. 2023;32:6706–22. 36970881 10.1111/jocn.16671

[16] GBD 2021 Causes of Death Collaborators. Global burden of 288 causes of death and life expectancy decomposition in 204 countries and territories and 811 subnational locations, 1990-2021: a systematic analysis for the Global Burden of Disease Study 2021. Lancet. 2024;403:2100–32. 38582094 10.1016/S0140-6736(24)00367-2 PMC11126520

[17] Tremblay D Touati N Roberge D. Understanding cancer networks better to implement them more effectively: a mixed methods multi-case study. Implement Sci. 2016;11:39. 27000152 10.1186/s13012-016-0404-8 PMC4802906

[18] Feola S Chiaro J Fusciello M. PeptiVAX: a new adaptable peptides-delivery platform for development of CTL-based, SARS-CoV-2 vaccines. Int J Biol Macromol. 2024;262(Pt 1):129926. 38331062 10.1016/j.ijbiomac.2024.129926

[19] Allkja J Bjarnsholt T Coenye T. Minimum information guideline for spectrophotometric and fluorometric methods to assess biofilm formation in microplates. Biofilm. 2019;2:100010. 33447797 10.1016/j.bioflm.2019.100010 PMC7798448

[20] Tseng TJ Guo SE Hsieh HW Lo KW. The effect of a multidimensional teaching strategy on the self-efficacy and critical thinking dispositions of nursing students: a quasi-experimental study. Nurse Educ Today. 2022;119:105531. 36194970 10.1016/j.nedt.2022.105531

[21] Xie X Fu Q Bao Z Zhang Y Zhou D. Clinical value of different anti-D immunoglobulin strategies for preventing Rh hemolytic disease of the fetus and newborn: a network meta-analysis. PLoS One. 2020;15:e0230073. 32163467 10.1371/journal.pone.0230073 PMC7067404

[22] Kamdar M Solomon SR Arnason J; TRANSFORM Investigators. Lisocabtagene maraleucel versus standard of care with salvage chemotherapy followed by autologous stem cell transplantation as second-line treatment in patients with relapsed or refractory large B-cell lymphoma (TRANSFORM): results from an interim analysis of an open-label, randomised, phase 3 trial. Lancet. 2022;399:2294–308. 35717989 10.1016/S0140-6736(22)00662-6

[23] da Costa BR Pereira TV Saadat P. Effectiveness and safety of non-steroidal anti-inflammatory drugs and opioid treatment for knee and hip osteoarthritis: network meta-analysis. BMJ. 2021;375:n2321. 34642179 10.1136/bmj.n2321 PMC8506236

